# Diagnostic value of blood-derived microRNAs for schizophrenia: results of a meta-analysis and validation

**DOI:** 10.1038/s41598-017-15751-5

**Published:** 2017-11-10

**Authors:** Sha Liu, Fuquan Zhang, Xijin Wang, Yin Yao Shugart, Yingying Zhao, Xinrong Li, Zhifen Liu, Ning Sun, Chunxia Yang, Kerang Zhang, Weihua Yue, Xin Yu, Yong Xu

**Affiliations:** 1 Department of Psychiatry, First Hospital/First Clinical Medical College of Shanxi Medical University, Taiyuan, 030001 P. R. China; 2 Multi-Disciplinary Team (MDT) Center for Cognitive Impairment and Sleep Disorders, First Hospital of Shanxi Medical University, Taiyuan, 030001 P. R. China; 3 Wuxi Mental Health Center, Wuxi, 214000 P. R. China; 4 The First Psychiatric Hospital of Harbin, Harbin, 150056 P. R. China; 5 Unit on Statistical Genomics, Intramural Research Program, National Institute of Mental Health, National Institutes of Health, Bethesda, Maryland USA; 6 Depression Treatment Center, Beijing Anding Hospital of Capital Medical University, Beijing, 100088 P. R. China; 7 Institute of Mental Health, Peking University Sixth Hospital, Peking University, Beijing, 100191 P. R. China

## Abstract

There is an increasing interest in searching biomarkers for schizophrenia (SZ) diagnosis, which overcomes the drawbacks inherent with the subjective diagnostic methods. MicroRNA (miRNA) fingerprints have been explored for disease diagnosis. We performed a meta-analysis to examine miRNA diagnostic value for SZ and further validated the meta-analysis results. Using following terms: schizophrenia/SZ, microRNA/miRNA, diagnosis, sensitivity and specificity, we searched databases restricted to English language and reviewed all articles published from January 1990 to October 2016. All extracted data were statistically analyzed and the results were further validated with peripheral blood mononuclear cells (PBMNCs) isolated from patients and healthy controls using RT-qPCR and receiver operating characteristic (ROC) analysis. A total of 6 studies involving 330 patients and 202 healthy controls were included for meta-analysis. The pooled sensitivity, specificity and diagnostic odds ratio were 0.81 (95% CI: 0.75-0.86), 0.81 (95% CI: 0.72-0.88) and 18 (95% CI: 9-34), respectively; the positive and negative likelihood ratio was 4.3 and 0.24 respectively; the area under the curve in summary ROC was 0.87 (95% CI: 0.84-0.90). Validation revealed that miR-181b-5p, miR-21-5p, miR-195-5p, miR-137, miR-346 and miR-34a-5p in PBMNCs had high diagnostic sensitivity and specificity in the context of schizophrenia. In conclusion, blood-derived miRNAs might be promising biomarkers for SZ diagnosis.

## Introduction

MicroRNAs (miRNAs) are a class of small non-coding RNAs (approximately 19–22 nucleotides in length) that are generally believed to negatively regulate gene expression, thereby controlling a wide range of biological processes and functions^1,2^. They bind sequences in the target messenger RNA 3′-untranslated regions through complementarity and form RNA-RNA complex, leading eventually to mRNA degradation or translational inhibition^3,4^. It has been shown that microRNAs can also up-regulate translation depending on cellular context^5^. Additionally, miRNA regulation of gene expression by targeting the sequences outside the messenger RNA 3′-untranslated regions has also been reported^6,7^. Alternation of miRNA expression has been discovered in various human diseases and increasing evidence has indicated that miRNA fingerprints are useful biomarkers in disease diagnosis and prognosis prediction^8–18^.

Schizophrenia (SZ) is a debilitating mental disorder with an overall prevalence estimate of around 4.0 per 1,000 people^19^. The disease is characterized by early adulthood onset and complex clinical symptoms with the etiology remaining elusive^20^. In contemporary practice, diagnosis of schizophrenia is still based on clinical interviews and careful observations, which is subjective and can lead to mis-diagnosis and/or delay in diagnosis, necessitating the development of objective diagnostic methods^21–23^. A considerable number of studies have demonstrated altered gene/miRNA expression in various tissues from patients with schizophrenia^24–40^, leading to an extensive search for gene expression patterns and/or miRNA fingerprints as biomarkers for SZ diagnosis and treatment monitoring^33,37,39–49^.

In this study, we conducted a systematic review on findings in blood-derived miRNA expression in patients with SZ, and performed a meta-analysis to evaluate the diagnostic value of blood-derived miRNAs. Furthermore, using RT-qPCR and receiver operating characteristic (ROC) analysis, we validated 6 miRNAs identified in the meta-analysis with peripheral blood mononuclear cells (PBMNCs) isolated from 39 patients and 50 healthy controls.

## Materials and Methods

### Meta-analysis

#### Literature search

A systematic review of the literature published in English from January 1990 to October 2016 was performed. The databases that were searched included MEDLINE, EMBASE, the Cochrane Database of Systematic Reviews, the Cochrane Central Register of Controlled Trials (CENTRAL), Health Technology Assessment Database, and Web of Science. The terms relating to or describing blood-derived miRNA expression in patients with schizophrenia and exploration of miRNAs as diagnostic markers were used for the database search, which included schizophrenia/SZ, microRNA/miRNA, diagnosis, sensitivity and specificity.

The protocol of this study has been registered in the PROSPERO, an international database of prospectively registered systematic reviews in health and social care, with a registration No. CRD42016037976 (https://www.crd.york.ac.uk/PROSPERO/).

#### Criteria for study inclusion and exclusion

All literature describing both quantitative and qualitative studies that investigated miRNA expression in SZ and delineated the relationship between miRNAs expression and SZ were included, which could be original reports, letters, reviews, editorial articles, or conference abstracts.

All studies included in this meta-analysis met all of the following criteria: (1) they were either randomized trials or observational studies; (2) all SZ diagnoses were made following clinically recognized diagnostic criteria; (3) all studies evaluated miRNA expression as biomarkers for the diagnosis of SZ; (4) All studies provided information on sample sizes and contained sensitivity and specificity data. Studies without sensitivity and specificity data or overlapping reports using same subjects were excluded.

#### Data extraction and quality assessment

Using a standardized form, two reviewers independently assessed all studies that were chosen for the final meta-analysis. We extracted following information from those selected articles: the first author, publication year, sample size, specimen, miRNA expression results, sensitivity and specificity analysis data. The quality of methodologies of those included studies was determined by quality assessment of diagnostic accuracy studies (QUADAS), an evidence-based quality assessment tool for systematic reviews of diagnostic accuracy studies^50^.

#### Meta-analysis

Information of all miRNA changes in patients was retrieved from all selected studies and used for meta-analysis. The true positive (TP), false positive (FP), true negative (TN) and false negative (FN) were defined as follows: significant miRNA changes (either increase or decrease compared with healthy controls, p < 0.05) found in an individual clinically diagnosed with schizophrenia was taken as true positive; significant miRNA changes found in an individual clinically diagnosed with non-schizophrenia was taken as false positive; non-significant miRNA changes found in an individual clinically diagnosed with non-schizophrenia was taken as true negative; non-significant miRNA changes found in an individual clinically diagnosed with schizophrenia was taken as false negative. The TP, FP, TN and FN total numbers from all selected studies were used to construct 2 × 2 contingency tables as described elsewhere^51^, and then pooled sensitivity and specificity, positive likelihood ratio (PLR), negative likelihood ratio (NLR), diagnostic odds ratio (DOR), and their corresponding 95% confidence intervals (CI) were computed. Post-test probability calculations were done using the Fagan’s nomogram, the heterogeneity across studies was determined using I^2^ test and Galbraith plot, and publication bias was assessed using Deeks’ funnel plots. All analyses were performed using the Stata 12.0 software (the StataCorp, College Station, Texas, USA) and the Review Manager 5.3 (Cochrane Community, London, UK). Two-sided statistical tests were performed and a *p* value < 0.05 was considered statistically significant.

### Further validation of meta-analysis results

#### Subjects

A total of 39 patients with schizophrenia in acute stage who had been drug-free for at least 3 months were recruited between March 2015 and February 2016. Clinical diagnosis was performed by at least two consultant psychiatrists independently according to the Diagnostic and Statistical Manual of Mental Disorders Fourth Edition (DSM-IV) criteria for SZ (https://justines2010blog.files.wordpress.com/2011/03/dsm-iv.pdf), relying on the Chinese Version of the Modified Structured Clinical Interview for DSM-IV TR Axis I Disorders Patient Edition (SCID-I/P,11/2002 revision). Those who were pregnant or had significant medical conditions, unstable psychiatric features (e.g., suicidal feelings) or had a history of substance abuse or drug addiction within the previous 6 months were excluded. This study was approved by the Research Ethics Committee of the First Hospital of Shanxi Medical University. Written informed consent was obtained from healthy participants and a first-degree relative of patients. All participants were of unrelated Chinese Han nationality. To ensure adequate power to detect a pre-specified effect, the sample size was chosen using the Power and Sample Size Program (http://biostat.mc.vanderbilt.edu/PowerSampleSize). The control group consisted of 50 healthy volunteers who were recruited from local communities or during routine health check-ups. All control subjects were assessed using the SCID-I/P, 11/2002 revision. Subjects with diagnosed diseases or a history of major psychiatric disorders or suicidal behavior were excluded, and those who had a first-degree relative with a history of severe mental disorder or suicidal behavior were also excluded. All experiments were performed in accordance with the China’s Ministry of Health guidelines on biomedical research involving humans.

#### Analysis of miRNA expression in PBMNCs by reverse transcription-quantitative polymerase chain reaction (RT-qPCR)

The ROC curve is a primary tool for the evaluation of diagnostic tests. In a ROC curve, the sensitivity is plotted against the specificity and each point on the ROC curve represents a sensitivity/specificity pair. The area under the curve (AUC) is a measure of how well a parameter can distinguish between the diseased and the normal. Based on a satisfying AUC value, miR-137, miR-181b-5p, miR-21-5p, miR-195-5p, and miR-34a-5p were chosen for validation. Additionally, miR-346 was validated because its expression has been detected in multiple tissues^30,37^. Blood sample collection, PBMNC isolation, total RNA extraction were performed as described elsewhere^46^. RNA quantification was done using spectrophotometry, and RNA quality was evaluated by examining the ratios of OD_260_/OD_280_ and OD_260_/OD_230_. One µg of RNA was reverse transcribed using the miScript Reverse Transcription kit (Qiagen China Co. Ltd., Shanghai, China) according to the manufacturer’s instructions. RT primers are listed in Supplementary Table 1. Afterwards, 2 µl of RT product was used for measurement of miRNAs with qPCR using the miScript SYBR Green PCR kit (Qiagen China Co. Ltd.). The PCR primers were listed in Supplementary Table 2. PCR was performed on the 7900HT Sequence Detection System (Applied Biosystems) as follows, step 1: 95 °C for 15 min; and step 2: 94 °C for 15 s, 55 °C for 30 s and 70 °C for 34 s. Step 2 was repeated for 40 cycles. The relative expression level of each individual miRNA after normalization to U6 snRNA was calculated using the 2^−∆∆Ct^ method.

#### Receiver operating characteristic (ROC) curve analysis

ROC curves were drawn and the area under the curve (AUC) was measured to determine and compare the diagnostic values of miRNAs using the Statistical Package for Social Sciences version 17.0 (SPSS 17.0) (SPSS Inc., Chicago, IL, USA). The ROC curve creates a complete sensitivity/specificity report for a diagnostic test. In a ROC curve the true positive rate (sensitivity) is plotted against the false positive rate (specificity) for different cut-off points of a parameter, and the AUC shows how well a parameter can distinguish between diseased and normal groups.

#### Statistical analysis

MiRNA relative levels determined in the PBMNCs were tested for normality with the Shapiro-Wilk test followed by the Mann-Whitney test for significant differences. These tests were performed using the SPSS 17.0 software, and *p* < 0.05 was considered statistically significant. The predictive power of those 6 validated miRNAs together was analyzed with the leave-one-out support vector machine using the Matlab software (The MathWorks Inc., Natick, MA, USA). With this analysis, the diagnostic accuracy and other relevant data (the true positive, false positive, true negative and false negative) were generated.

## Results

### Systematic review and meta-analysis

**Figure 1:**
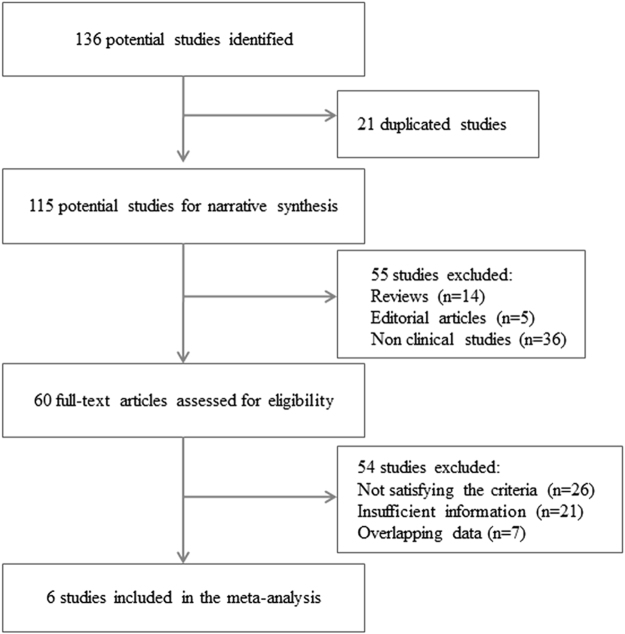
Process of study selection. A total of 136 studies were retrieved from the databases searched. Six articles were finally selected for a meta-analysis.

A total of 136 articles were retrieved, and 6 were finally selected for a meta-analysis (Fig. 1). All 6 articles were about case-control studies involving 330 patients and 202 healthy controls. Alternation of miRNA expression in serum (1 report) and PBMNCs (5 reports) were discovered. The details of these studies were summarized in Supplementary Table 3.

**Figure 2:**
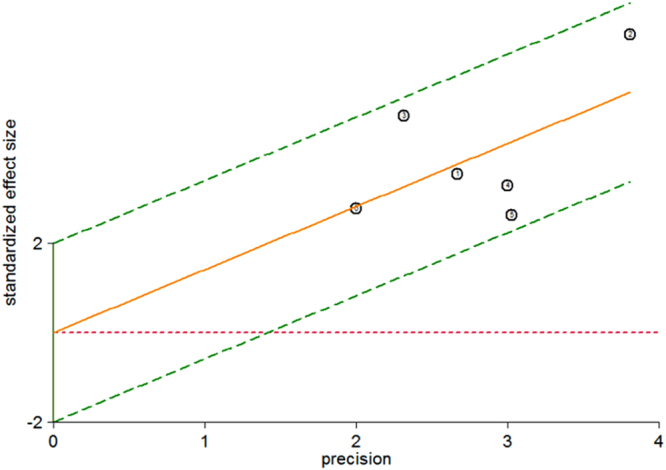
Galbraith plot for analysis of heterogeneity across studies. The yellow line depicted the regression line, parallel to the regression line, at a 2-standard-deviation distance, 2 lines (dotted green lines) created an interval in which all 6 studies (in small circles) fell, indicating no inconsistency was found across studies.

**Figure 3:**
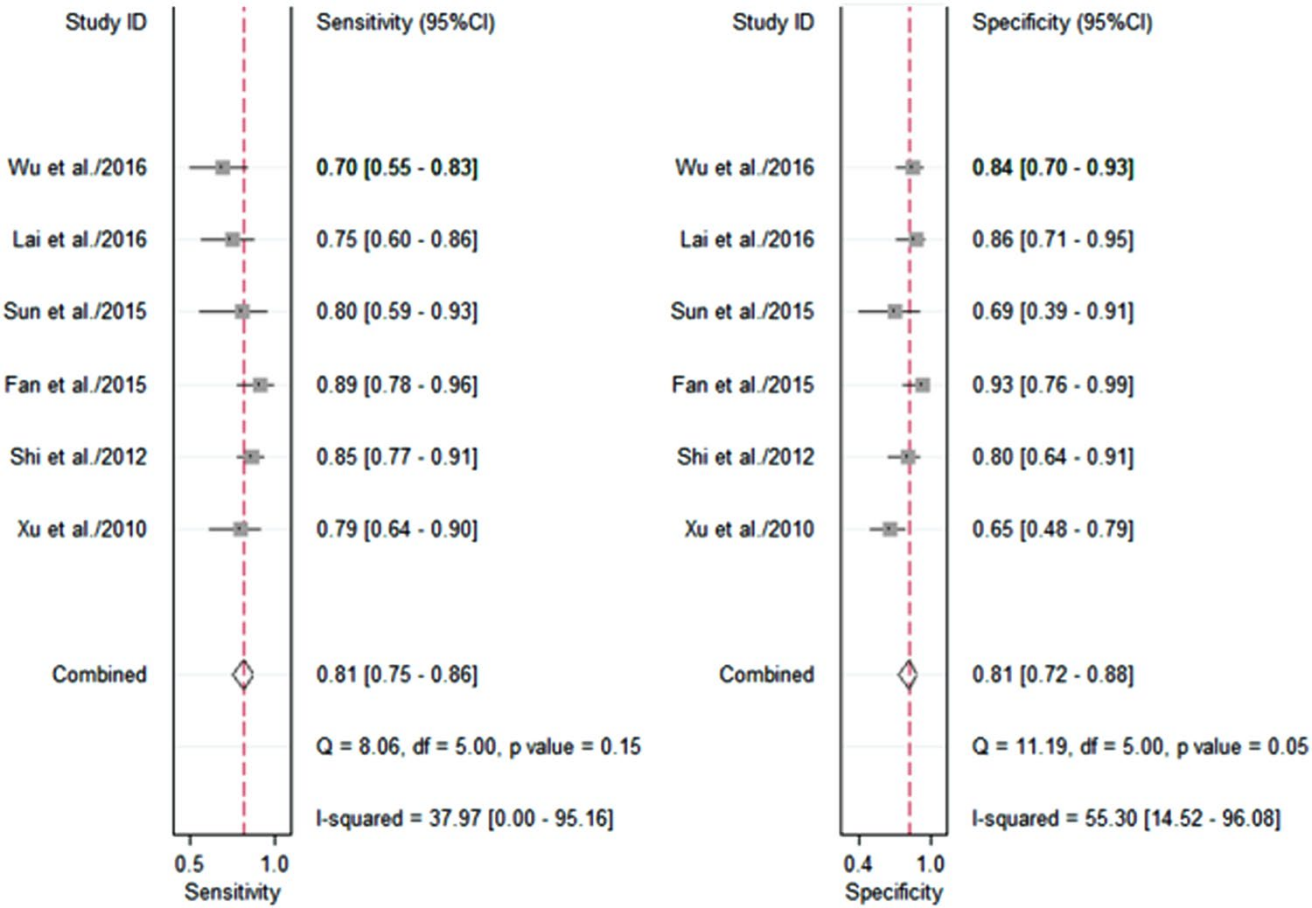
Pooled sensitivity and specificity. Shown in the left panel are sensitivity of each individual study and the pooled sensitivity, while shown in the right panel are specificity of each individual study and the pooled specificity.

**Figure 4:**
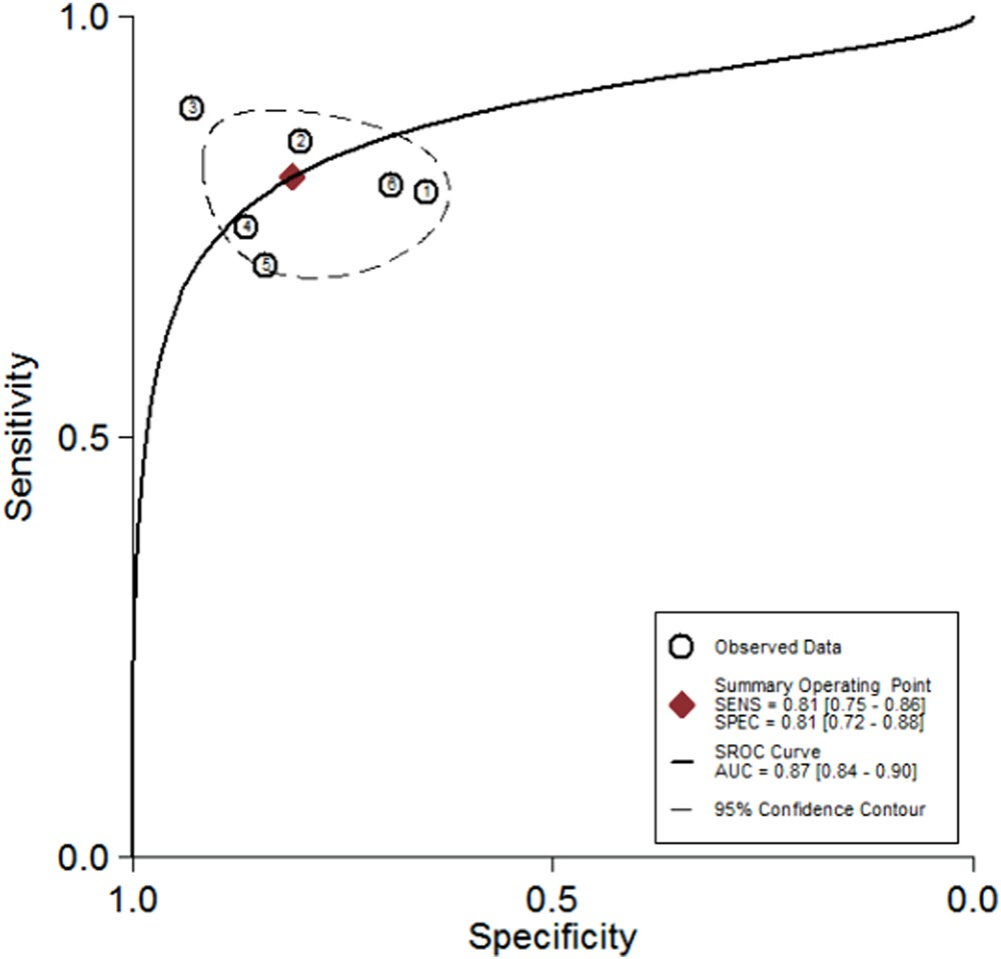
Summary receiver operating characteristic curve analysis. This figure showed that the area under the ROC curve is 0.87 (the point marked as red rhombus), indicating high accuracy of miRNAs as diagnostic markers for SZ.

**Figure 5:**
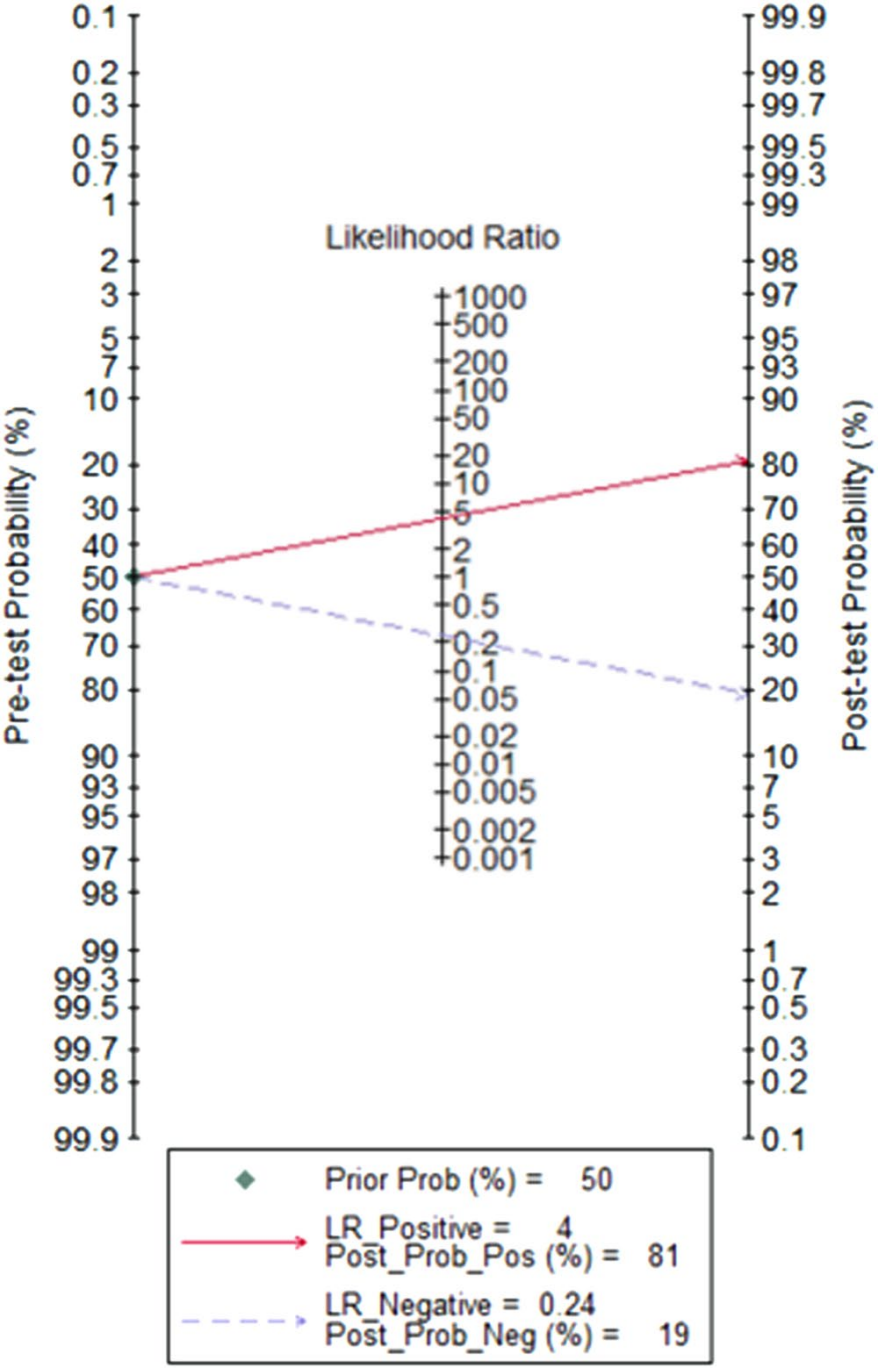
Fagan’s Nomogram for assessment of post-test probabilities. Fagan’s Nomogram showed that the positive likelihood ratio (PLR) was 4.3 and the negative likelihood ratio (NLR) was 0.24.

The heterogeneity I^2^ value for inter-study variability is 0 (95% CI: 0-100). Galbraith plot for heterogeneity analysis showed that there was no inconsistence across studies (Fig. 2). Analysis of miRNAs as diagnostic markers for SZ showed that the pooled sensitivity was 0.81 (95% CI: 0.75-0.86, I^2^ = 37.97%, *p* = 0.15) and the pooled specificity was 0.81 (95% CI: 0.72-0.88, I^2^ = 55.30%, *p* = 0.05) (Fig. 3). The area under the summary receiver operating characteristic curve was 0.87 (95% CI: 0.84-0.90) (Fig. 4). The AUC of 0.87 indicates good diagnostic accuracy, as the maximum AUC value is 1 meaning a 100% diagnostic accuracy for a test, while AUC = 0 suggests that the test has a zero accuracy, which however is extremely unlikely to happen in clinical practice. The PLR was 4.3 (95% CI: 2.8-6.5) and the NLR is 0.24 (95% CI: 20.17-0.32), as demonstrated in the Fagan’s nomogram in Fig. 5. Moreover, the combined diagnostic odds ratio (DOR) was 18 (95% CI: 9-34). DOR is the ratio of the odds of positivity in subjects with disease relative to the odds in subjects without disease. It combines sensitivity and specificity, thus DOR, as a single indicator, can be used to evaluate the accuracy of a test, and a DOR value > 1.0 indicates the test has discriminatory ability (to discriminate subjects with disease from subjects without disease).

**Figure 6:**
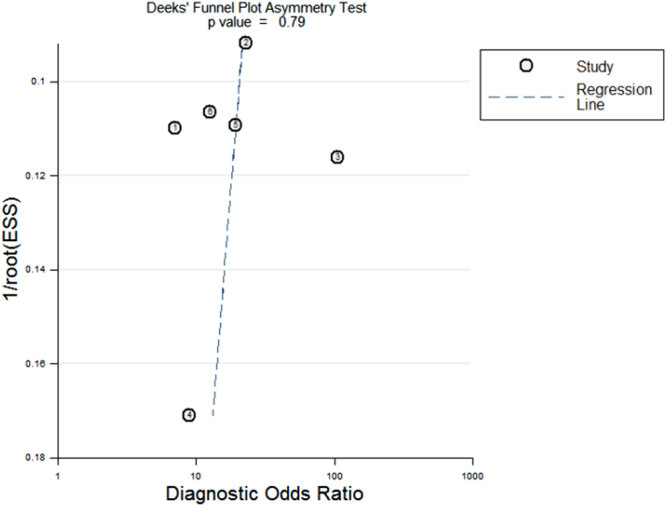
Funnel plot for the assessment of publication bias. The funnel plot as shown here appeared approximately symmetrical around the combined diagnostic odds ratio of 18. The Deek’s test further confirmed no publication bias (*p* = 0.79).

Funnel plot analysis showed that the plot appeared approximately symmetrical around the combined diagnostic odds ratio of 18 (Fig. 6), indicating a lack of publication bias, which was further confirmed by the Further Deeks’ test (*p* = 0.79, Fig. 6).

### Validation

**Figure 7:**
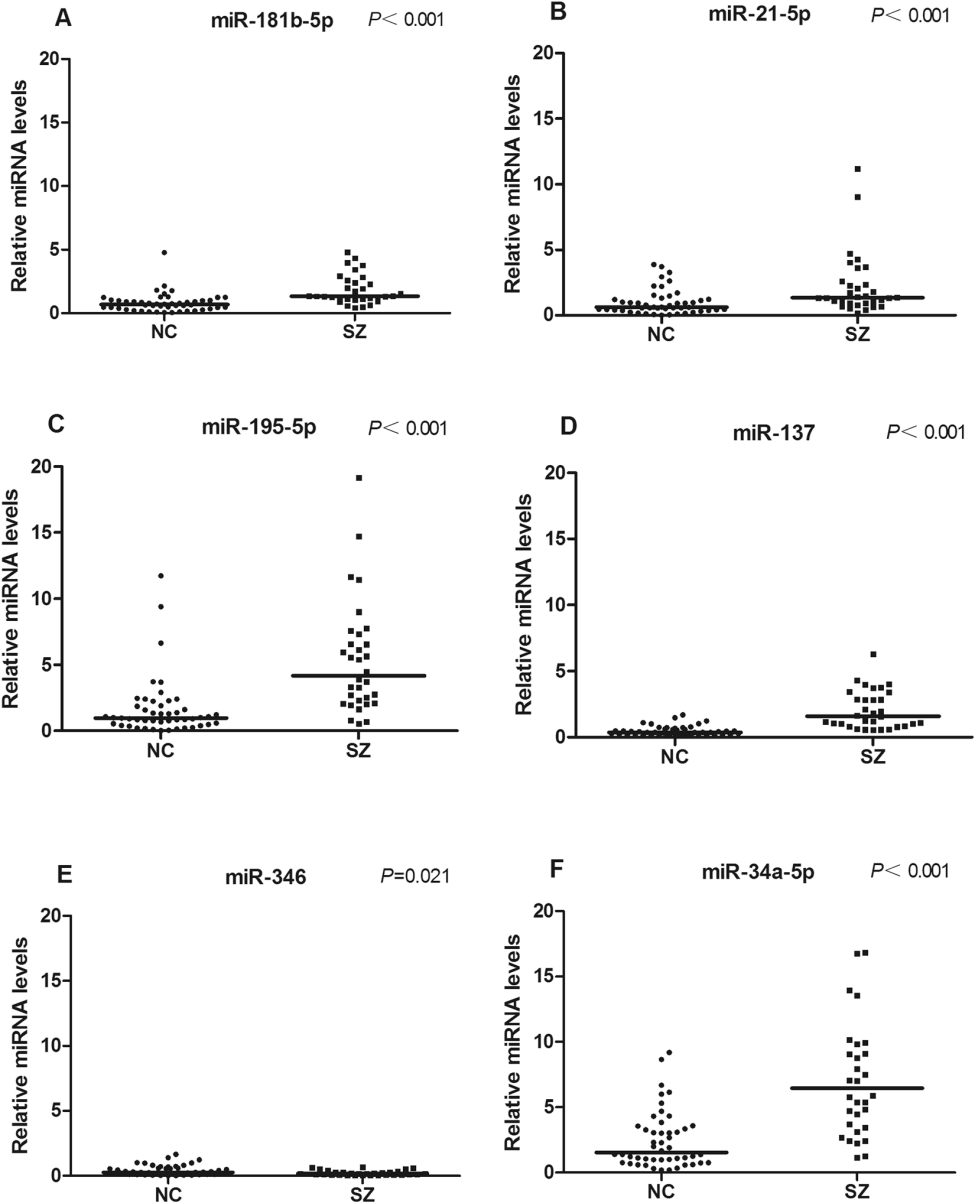
MiRNA validation results. The relative expression levels of 6 miRNAs in PBMNCs from normal controls (NC) (n = 50) and patients with schizophrenia (SZ) (n = 39) are shown as indicated. Each dot represents the relative miRNA level of a subject. The vertical line indicates the median. The Mann-Whitney test was applied for analysis of difference significance between the patients and healthy controls. *p* < 0.05 was considered statistically significant.

**Figure 8:**
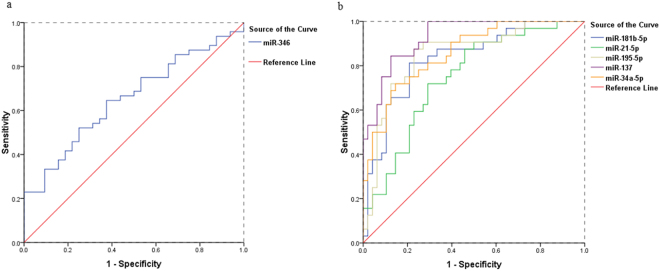
ROC curve analysis of validation data. Figure 8a is the ROC curve for miR-346 which was reduced in patients. Figure 8b include ROC curves for miR-181b-5p, miR-21-5p, miR-195-5p, miR-137 and miR-34a-5p which were increased in patients. The sensitivity, specificity and AUC value of each miRNA in SZ diagnosis are listed in Supplementary Table 6.

To validate the findings from the meta-analysis, 39 patients and 50 healthy individuals were recruited. Over half of patients (20/39) had first-episode schizophrenia. The summarized demographics of all participants are listed in Supplementary Table 4. Levels of miR-181b-5p, miR-21-5p, miR-195-5p, miR-137, miR-346 and miR-34a-5p in PBMNCs differ significantly between patients and controls, which are shown in Fig. 7 as well as in Supplementary Table 5. As the Shapiro-Wilk test showed non-normal distribution of the data, the Mann-Whitney test was applied for analysis of difference significance (Fig. 7 and Supplementary Table 5). Diagnostic value of each of these 6 miRNAs was analyzed by ROC (Fig. 8), and the diagnostic sensitivity, specificity and AUC are listed in Supplementary Table 6. Furthermore, the leave-one-out support vector machine analysis showed that those 6 miRNAs detected in the validation study together had a diagnostic accuracy of 86.37%, true positive of 81.76%, true negative of 93.43%, false positive of 8.24% and false negative of 6.57%. Our validation results aligned well with the meta-analysis data (Figs 3 and 4), suggesting PBMNC miRNAs as biomarkers for SZ diagnosis have high diagnostic accuracy.

## Discussion

There have been no objective diagnostic tools for SZ, and SZ diagnosis still relies on subjective analysis. Recently, molecular examinations have discovered aberrant gene expression including miRNAs in different sources of samples, e.g., the brain tissue, plasma, serum and PBMNCs, derived from patients with SZ^24–40^, raising the possibility that certain gene expression patterns distinctively present in patients might be useful for SZ diagnosis and/or treatment monitoring^33,37,39,40,42–49^. Peripheral blood, due to its accessibility and ease in procurement, has become a particularly focused source for search of diagnostic biomarkers for SZ^23^. In this study, we first systematically reviewed the literature and performed a meta-analysis to evaluate blood-derived miRNAs as potential biomarkers for SZ diagnosis. We showed that miRNAs had high pooled sensitivity and specificity, i.e., 0.80 and 0.87 respectively with an AUC of 0.87 in SZ diagnosis. As analysis revealed absence of publication bias and no inconsistence across studies, strengthening the validity of the findings.

Among 6 papers finally selected for meta-analysis, 5 reported miRNA expression changes in PBMNCs and one revealed abnormal miRNA levels in serum. Based on this, next, using RT-qPCR and ROC analysis, we validated 6 miRNAs in PBMNCs isolated from 39 patients and 50 healthy controls. The results showed that miR-346 was significantly reduced while the other 5 miRNAs were remarkably increased in PBMNCs from patients, which aligns with the findings reported in the 5 studies (Supplementary Table 3). The ROC analysis demonstrated diagnostic sensitivity ranging from 0.646–0.875, and specificity ranging from 0.625–0.875 with an AUC of 0.653–0.928 for the 6 miRNAs, also in agreement with those reported previously (Supplementary Table 3). Further analysis with the support vector machine revealed that those 6 miRNAs together had a high diagnostic accuracy, although a negative control miRNA that does not change between patients and healthy subjects was not included for analysis, which is a limitation of this study. Of note, the miR-137 locus has been linked to schizophrenia, but in post-mortem analysis of brain tissue, miR-137 has not been shown to be differentially expressed in schizophrenia^52^, which contrasts with the finding in PBMNCs. The reason for such discrepancy, we speculated, might be that miR-137 dysregulation in PBMNCs is the results of the disease. We focused on the assessment of blood-derived miRNAs (it turned out to be mostly PBMNC-derived as reported in literature) as biomarkers for schizophrenia diagnosis, and whether PBMNC mRNA changes contribute to pathophysiology of schizophrenia is outside the scope of this study. Because schizophrenia is a disease of the central nervous system, we believe the major players exist in the nervous system. We strongly felt that abnormal PBMNC miRNA levels in patients are not the cause but rather a result of schizophrenia, which however remains to be elucidated.

To avoid the confounding effect from medications, we recruited patients who had not received pharmacologic treatment for at least 3 months and collected blood samples prior to pharmacologic intervention. Although in recent years the public have gained a better understanding of schizophrenia, being with schizophrenia is still widely deemed a stigma among patient families as well as in the public in China. As a result, patients and their families are often reluctant to see healthcare providers, delaying pharmacologic treatment. In this study, we did not have much difficulty recruiting patients who met the drug-free requirement.

Schizophrenia and bipolar disorder are often misdiagnosed as one another, as these two diseases share a range of similar clinical manifestations. Additionally, some schizophrenia patients have depressive symptoms, and it can be difficult to differentiate schizophrenia with significant depressive symptoms from major depressive disorder with psychotic features. Therefore, specific biomarkers for differentiation of these disorders are particularly desirable. In a case-control study, Belzeaux *et al*. performed an array analysis and found that in PBMNCs 9 miRNAs, i.e., miR-589, miR-579, miR-941, miR-133a, miR-494, miR-107, miR-148a, miR-652 and miR-425-3p were elevated and 5 (miR-517b, miR-636, miR-1243, miR-381 and miR-200c) were reduced in major depressive patients (Supplementary Table 7)^53^. Maffioletti *et al*. showed in an array assay of peripheral whole blood that 5 miRNAs (let-7a-5p, let-7d-5p, let-7f-5p, miR-24-3p and miR-425-3p) were specifically altered in major depression patients and 5 (miR-140-3p, miR-30d-5p, miR-330-5p, miR-378a-5p and miR-21-3p) in bipolar disorder patients, whereas 2 miRNAs (miR-330-3p and miR-345-5p) were dysregulated in both the diseases (Supplementary Table 7)^54^. Using real-time RT-PCR method, Rong *et al*. discovered that patients with bipolar disorder had a markedly lower level of plasma miR-134 levels than healthy subjects^55^. These findings indeed reveal distinct blood miRNA signatures (Supplementary Table 7), suggesting miRNA changes in blood are useful in specific diagnosis of SZ, bipolar disorder and major depressive disorder.

Use of biomarkers for SZ diagnosis could overcome the drawbacks inherent with subjective diagnostic methods, which is therefore desirable. The meta-analysis and validation results suggest blood-derived miRNAs, especially those from PBMNCs are promising biomarkers for SZ diagnosis. Several limitations of this study should be acknowledged. First, only 6 studies were included for meta-analysis. Second, those 6 studies utilized different methods for quantitative detection and normalization in miRNA analysis, which may cause different results across studies, although not detected. Finally, the relatively small sample size of each study may limit the statistical power.

In conclusion, measurement of blood-derived miRNA levels might be a promising objective method for SZ diagnosis. Prospective, multi-centered, large-scale studies are warranted.

## Electronic supplementary material

Supplementary tables

## References

[1] Ambros V (2004). The functions of animal microRNAs. Nature..

[2] Bartel DP (2009). MicroRNAs: Target recognition and regulatory functions. Cell..

[3] Djuranovic S, Nahvi A, Green R (2012). miRNA-mediated gene silencing by translational repression followed by mRNA deadenylation and decay. Science..

[4] Jonas S, Izaurralde E (2015). Towards a molecular understanding of microRNA-mediated gene silencing. Nat Rev Genet..

[5] Vasudevan S, Tong Y, Steitz JA (2007). Switching from repression to activation: microRNAs can up-regulate translation. Science..

[6] Hausser J, Syed AP, Bilen B, Zavolan M (2013). Analysis of CDS-located miRNA target sites suggests that they can effectively inhibit translation. Genome Res..

[7] Tay Y, Zhang J, Thomson AM, Lim B, Rigoutsos I (2008). MicroRNAs to Nanog, Oct4 and Sox2 coding regions modulate embryonic stem cell differentiation. Nature..

[8] Tomasetti M, Lee W, Santarelli L, Neuzil J (2017). Exosome-derived microRNAs in cancer metabolism: possible implications in cancer diagnostics and therapy. Exp Mol Med..

[9] Viereck J, Thum T (2017). Circulating Noncoding RNAs as Biomarkers of Cardiovascular Disease and Injury. Circ Res..

[10] Güçlü A (2017). MicroRNA-125b as a new potential biomarker on diagnosis of renal ischemia-reperfusion injury. J Surg Res..

[11] Troiano G (2016). Circulating miRNAs from blood, plasma or serum as promising clinical biomarkers in oral squamous cell carcinoma: A systematic review of current findings. Oral Oncol..

[12] Martirosyan NL (2016). The Role of microRNA markers in the diagnosis, treatment, and outcome prediction of spinal cord injury. Front Surg..

[13] Zhang L, Cao D, Tang L, Sun C, Hu Y (2016). A panel of circulating miRNAs as diagnostic biomarkers for screening multiple myeloma: a systematic review and meta-analysis. Int J Lab Hematol..

[14] Hicks SD, Middleton FA (2016). A comparative review of microRNA expression patterns in autism spectrum disorder. Front Psychiatry..

[15] Martinez B, Peplow PV (2016). Blood microRNAs as potential diagnostic and prognostic markers in cerebral ischemic injury. Neural Regen Res..

[16] Alipoor SD (2016). The roles of miRNAs as potential biomarkers in lung diseases. Eur J Pharmacol..

[17] Lai CY (2011). MicroRNA expression aberration as potential peripheral blood biomarkers for schizophrenia. PLoS One..

[18] Sun XY (2015). Aberrant microRNA expression in peripheral plasma and mononuclear cells as specific blood-based biomarkers in schizophrenia patients. J Clin Neurosci..

[19] McGrath J, Saha S, Chant D, Welham J (2008). Schizophrenia: a concise overview of incidence, prevalence, and mortality. Epidemiol Rev..

[20] Sullivan PF (2005). The genetics of schizophrenia. PLoS Med..

[21] Weickert CS, Weickert TW, Pillai A, Buckley PF (2013). Biomarkers in schizophrenia: a brief conceptual consideration. Dis Markers..

[22] Pickard BS (2015). Schizophrenia biomarkers: translating the descriptive into the diagnostic. J Psychopharmacol..

[23] Lai CY (2016). Biomarkers in schizophrenia: A focus on blood based diagnostics and theranostics. World J Psychiatry..

[24] Perkins DO (2007). microRNA expression in the prefrontal cortex of individuals with schizophrenia and schizoaffective disorder. Genome Biology..

[25] Beveridge NJ (2008). Dysregulation of miRNA 181b in the temporal cortex in schizophrenia. Human molecular genetics..

[26] Mellios. N (2012). Gender-specific reduction of estrogen-sensitive small RNA, miR-30b, in subjects with schizophrenia. Schizophrenia bulletin..

[27] Xu Y (2016). Exploring transcription factors-microRNAs co-regulation networks in schizophrenia. Schizophrenia bulletin..

[28] Burmistrova OA (2007). MicroRNA in schizophrenia: genetic and expression analysis of miR-130b (22q11). Biochemistry (Mosc)..

[29] Mellios N (2009). Molecular determinants of dysregulated GABAergic gene expression in the prefrontal cortex of subjects with schizophrenia. Biol Psychiatry..

[30] Zhu Y, Kalbfleisch T, Brennan MD, Li Y (2009). A MicroRNA gene is hosted in an intron of a schizophrenia-susceptibility gene. Schizophr Res..

[31] Beveridge NJ, Gardiner E, Carroll AP, Tooney PA, Cairns MJ (2010). Schizophrenia is associated with an increase in cortical microRNA biogenesis. Mol Psychiatry..

[32] Kim AH (2010). MicroRNA expression profiling in the prefrontal cortex of individuals affected with schizophrenia and bipolar disorders. Schizophr Res..

[33] Xu Y (2010). MicroRNAs and target site screening reveals a pre-microRNA-30e variant associated with schizophrenia. Schizophr Res..

[34] Moreau MP, Bruse SE, David-Rus R, Buyske S, Brzustowicz LM (2011). Altered microRNA expression profiles in postmortem brain samples from individuals with schizophrenia and bipolar disorder. Biol Psychiatry..

[35] Santarelli DM, Beveridge NJ, Tooney PA, Cairns MJ (2011). Upregulation of dicer and microRNA expression in the dorsolateral prefrontal cortex Brodmann area 46 in schizophrenia. Biol Psychiatry..

[36] Miller BH (2012). MicroRNA-132 dysregulation in schizophrenia has implications for both neurodevelopment and adult brain function. Proc Natl Acad Sci USA.

[37] Shi W (2012). Aberrant expression of serum miRNAs in schizophrenia. J Psychiatr Res..

[38] Banigan MG (2013). Differential expression of exosomal microRNAs in prefrontal cortices of schizophrenia and bipolar disorder patients. PLoS One..

[39] Fan HM (2015). Altered microRNA expression in peripheral blood mononuclear cells from young patients with schizophrenia. J Mol Neurosci..

[40] Sun XY (2015). Aberrant microRNA expression in peripheral plasma and mononuclear cells as specific blood-based biomarkers in schizophrenia patients. Journal of clinical neuroscience..

[41] Wei H (2015). Detection of circulating miRNA levels in schizophrenia. Am J Psychiatry..

[42] Lai CY (2016). Aberrant expression of microRNAs as biomarker for schizophrenia: from acute state to partial remission, and from peripheral blood to cortical tissue. Transl Psychiatry..

[43] Wu S (2016). MicroRNA-137 inhibits EFNB2 expression affected by a genetic variant and is expressed aberrantly in peripheral blood of schizophrenia patients. EBioMedicine..

[44] Gardiner E (2012). Imprinted DLK1-DIO3 region of 14q32 defines a schizophrenia-associated miRNA signature in peripheral blood mononuclear cells. Mol psychiatry..

[45] Fernandes BS (2015). Peripheral brain-derived neurotrophic factor in schizophrenia and the role of antipsychotics: meta-analysis and implications. Mol psychiatry..

[46] Yao Y, Schroder J, Karlsson H (2008). Verification of proposed peripheral biomarkers in mononuclear cells of individuals with schizophrenia. Journal of psychiatric research..

[47] Mehler-Wex C (2006). Increased mRNA levels of the mitochondrial complex I 75-kDa subunit. A potential peripheral marker of early onset schizophrenia?. European child & adolescent psychiatry..

[48] Dror N (2002). State-dependent alterations in mitochondrial complex I activity in platelets: a potential peripheral marker for schizophrenia. Mol psychiatry..

[49] Liu S (2017). The early growth response protein 1-miR-30a-5p-neurogenic differentiation factor 1 axis as a novel biomarker for schizophrenia diagnosis and treatment monitoring. Transl Psychiatry..

[50] Whiting PF (2006). Evaluation of QUADAS, a tool for the quality assessment of diagnostic accuracy studies. BMC medical research methodology..

[51] Hu YB (2016). Diagnostic value of microRNA for Alzheimer’s disease: a systematic review and meta-analysis. Front Aging Neurosci..

[52] Mahmoudi E, Cairns MJ (2017). MiR-137: an important player in neural development and neoplastic transformation. Mol Psychiatry..

[53] Belzeaux R (2012). Responder and nonresponder patients exhibit different peripheral transcriptional signatures during major depressive episode. Transl Psychiatry..

[54] Maffioletti E (2016). Peripheral whole blood microRNA alterations in major depression and bipolar disorder. J Affect Disord..

[55] Rong H (2011). MicroRNA-134 plasma levels before and after treatment for bipolar mania. J Psychiatr Res..

