# Supplementary material for: Diagnostic value of blood-derived microRNAs for schizophrenia: results of a meta-analysis and validation
Source: Sci Rep. 2017 Nov 10;7:15328. doi: 10.1038/s41598-017-15751-5 (PMC5681644; doi:10.1038/s41598-017-15751-5)
Supplement: Supplementary file 1 — Supplementary tables [file 41598_2017_15751_MOESM1_ESM.pdf]

Title: Diagnostic value of blood-derived microRNAs for schizophrenia: results of a meta-analysis and validation

Authors: Sha Liu, Fuquan Zhang, Xijin Wang, Yin Yao Shugart, Yingying Zhao, Xinrong Li, Zhifen Liu, Ning Sun, Chunxia Yang, Kerang Zhang, Weihua Yue, Xin Yu, Yong Xu

Supplementary table 1. RT primers of miRNAs.

miRNA	RT primers (5'-3')
hsa-miR-181b-5p	GTCGTATCCAGTGCGTGTCGTGGAGTCGGCAATTGCACTGGATACGACACCCAC
hsa-miR-21-5p	GTCGTATCCAGTGCGTGTCGTGGAGTCGGCAATTGCACTGGATACGACTCAACA
hsa-miR-195-5p	GTCGTATCCAGTGCGTGTCGTGGAGTCGGCAATTGCACTGGATACGACGCCAAT
hsa-miR-137	GTCGTATCCAGTGCGTGTCGTGGAGTCGGCAATTGCACTGGATACGACCTACGC
hsa-miR-346	GTCGTATCCAGTGCGTGTCGTGGAGTCGGCAATTGCACTGGATACGACAGAGGC
hsa-miR-34a-5p	GTCGTATCCAGTGCGTGTCGTGGAGTCGGCAATTGCACTGGATACGACACAACC
U6	CGCTTCACGAATTTGCGTGTCAT

U6 is the internal reference. RT = reverse transcription.

Supplementary table 2. Real-time PCR primers of miRNAs.

miRNA		Real-time PCR primers (5'-3')
hsa-miR-181b-5p	F:	GGGAACATTCATTGCTGTCG
	R:	GTGCGTGTCGTGGAGTCG
hsa-miR-21-5p	F:	GGGTAGCTTATCAGACTGA
	R:	GTGCGTGTCGTGGAGTCG
hsa-miR-195-5p	F:	GGGTAGCAGCACAGAAAT
	R:	GTGCGTGTCGTGGAGTCG
hsa-miR-137	F:	GGGTTATTGCTTAAGAATAC
	R:	GTGCGTGTCGTGGAGTCG
hsa-miR-346	F:	GGGTGTCTGCCCGCATGCCT
	R:	GTGCGTGTCGTGGAGTCG
hsa-miR-34a-5p	F:	GGGTGGCAGTGTCTTAGCT
	R:	GTGCGTGTCGTGGAGTCG
U6	F:	GCTTCGGCAGCACATATACTAAAAT
	R:	CGCTTCACGAATTTGCGTGTCAT

F = forward primer, R = reverse primer.

Supplementary table 3. Summary of included studies in meta-analysis.

Study ID	Authors	Sample size (SZ vs. NC)	Specimen	AUC (95% CI)	SEN	SPE	TP a	FP b	FN c	TN d	QUADAS	miRNAs profile
1	Xu et al. 2010[33]	43 vs. 40	PBMC	0.74 (0.61-0.87)	80.0%	64.2%	34	14	9	26	11	miR-30e
2	Shi et al.2012[37]	115 vs. 40	Serum	0.85 (0.78-0.91)	85.0%	80.7%	98	8	17	32	11	miR-181b-5p, miR-219-2-3p, miR-195-5p, miR-346, miR-1308, let-7g
3	Fan, et al. 2015[39]	55 vs. 28	PBMC	0.97 (0.95-1.00)	89.3%	94.6%	49	2	6	26	12	miR-1273d, miR-1303, miR-21-5p, miR-3064-5p, miR-3131, miR-3687, miR-4428, miR-4725-3p, miR-5096
4	Sun et al. 2015[40]	25 vs. 13	PBMC	0.76 (0.58-0.93)	81.8%	68.0%	20	4	5	9	10	miR-30e
5	Lai et al. 2016[42]	48 vs. 37	PBMC	0.80 (0.69-0.93)	75.7%	86.5%	36	5	12	32	11	miR-34a-5p, miR-449a,miR-564, miR-432, miR-548d, miR-572, miR-652
6	Wu et al. 2016[43]	44 vs. 44	PBMC	0.80 (0.70-0.89)	70.5%	84.1%	31	7	13	37	13	miR-137
	Total	330 vs. 202										

AUC = the area under the curve, SEN = Sensitivity, SPE = Specificity, TP = true positives, FP = false positives, TN = true negatives, FN = false negatives, NA = not available, QUADAS = Quality Assessment of Diagnostic Accuracy Studies.

Supplementary table 4. The demographic characteristics of subjects.

	SZ	HC	*P* value
Age (years)	36.4 ± 9.7	36.0 ± 7.3	0.921
Males, n (%)	15 (38.9%)	18 (36%)	0.834
Age of onset (years)	28.2 ± 10.0		

SZ = schizophrenia patients, HC = healthy controls. Age was expressed as mean ± standard deviation.

Supplementary table 5. Relative miRNA levels in PBMNCs from controls and SZ patients.

MiRNAs	HC (n=50)	SZ (n=39)	*P*
miR-181b-5p	0.811±0.108	1.854±0.187	< 0.001
miR-21-5p	0.987±0.135	2.237±0.384	0.008
miR-195-5p	1.662±0.315	5.350±0.678	< 0.001
miR-137	0.469±0.052	2.079±0.231	< 0.001
miR-346	0.414±0.054	0.221±0.030	0.004
miR-34a-5p	2.465±0.307	8.174±1.064	< 0.001

All data were expressed as mean ± standard error of the mean.

Supplementary table 6. The diagnostic value of 6 miRNAs in PBMNCs for SZ.

MiRNAs	Sen	Spe	Youden index	AUC	P-value	95% CI
miR-181b-5p	0.813	0.792	0.605	0.831	<0.001	0.740-0.923
miR-21-5p	0.719	0.708	0.427	0.746	<0.001	0.639-0.854
miR-195-5p	0.875	0.771	0.646	0.848	<0.001	0.759-0.937
miR-137	0.844	0.875	0.719	0.928	<0.001	0.877-0.980
miR-34a-5p	0.719	0.854	0.573	0.857	<0.001	0.777-0.938
miR-346	0.646	0.625	0.271	0.653	0.021	0.534-0.772

Sen = sensitivity, Spe = specificity.

Supplementary table 7. Altered miRNAs in PBMNCs among SZ, bipolar disorder and major depressive disorder.

Schizophrenia	Bipolar disorder	Major depressive disorder
miR-30e	miR-140-3p	miR-589
miR-181b-5p	miR-30d-5p	miR-579
miR-219-2-3p	miR-330-5p	miR-941
miR-195-5p	miR-378a-5p	miR-133a
miR-346	miR-21-3p	miR-494
miR-1308	miR-330-3p	miR-107
let-7g	miR-345-5p	miR-148a
miR-1273d	miR-134	miR-652
miR-1303		miR-425-3p
miR-21-5p		miR-330-3p
miR-3064-5p		miR-345-5p
miR-3131		miR-24-3p
miR-3687		miR-425-3p
miR-4428		let-7a-5p
miR-4725-3p		let-7d-5p
miR-5096		let-7f-5p
miR-34a-5p		miR-517b
miR-449a		miR-636
miR-564		miR-1243
miR-432		miR-381
miR-548d		miR-200c
miR-572		
miR-652		
miR-137		
